# Tau positron emission tomography, cerebrospinal fluid and plasma biomarkers of neurodegeneration, and neurocognitive testing: an exploratory study of participants with myotonic dystrophy type 1

**DOI:** 10.1007/s00415-022-10970-x

**Published:** 2022-02-01

**Authors:** Robert Jr Laforce, Caroline Dallaire-Théroux, Annie M. Racine, Gersham Dent, Cristian Salinas-Valenzuela, Elizabeth Poulin, Anne-Marie Cayer, Daphnée Bédard-Tremblay, Thierry Rouleau-Bonenfant, Frédéric St-Onge, Susanna Schraen-Maschke, Jean-Mathieu Beauregard, Nicolas Sergeant, Jack Puymirat

**Affiliations:** 1 Clinique Interdisciplinaire de Mémoire, CHU de Québec, Québec, QC Canada; 2 Biogen Inc, Cambridge, MA USA; 3 Université de Lille, Inserm UMRS1172, CHU Lille, Lille, France; 4 Alzheimer & Tauopathies, LabEx DISTALZ, Lille, France

**Keywords:** Tau PET, Imaging, CSF, Cognition, Social cognition, Myotonic dystrophy type 1

## Abstract

**Objective:**

To investigate Tau pathology using multimodal biomarkers of neurodegeneration and neurocognition in participants with myotonic dystrophy type 1 (DM1).

**Methods:**

We recruited twelve participants with DM1 and, for comparison, two participants with Alzheimer’s Disease (AD). Participants underwent cognitive screening and social cognition testing using the Dépistage Cognitif de Québec (DCQ), among other tests. Biomarkers included Tau PET with [18F]-AV-1451, CSF (Aβ, Tau, phospho-Tau), and plasma (Aβ, Tau, Nf-L, GFAP) studies.

**Results:**

Of the twelve DM1 participants, seven completed the full protocol (Neurocognition 11/12; PET 7/12, CSF 9/12, plasma 12/12). Three DM1 participants were cognitively impaired (CI). On average, CI DM1 participants had lower scores on the DCQ compared to cognitively unimpaired (CU) DM1 participants (75.5/100 vs. 91.4/100) and were older (54 vs. 44 years old) but did not differ in years of education (11.3 vs. 11.1). The majority (6/7) of DM1 participants had no appreciable PET signal. Only one of the CI participants presented with elevated Tau PET SUVR in bilateral medial temporal lobes. This participant was the eldest and most cognitively impaired, and had the lowest CSF Aβ 1-42 and the highest CSF Tau levels, all suggestive of co-existing AD. CSF Tau and phospho-Tau levels were higher in the 3 CI compared to CU DM1 participants, but with a mean value lower than that typically observed in AD. Nf-L and GFAP were elevated in most DM1 participants (9/11 and 8/11, respectively). Finally, CSF phospho-Tau was significantly correlated with plasma Nf-L concentrations.

**Conclusions and relevance:**

We observed heterogenous cognitive and biomarker profiles in individuals with DM1. While some participants presented with abnormal PET and/or CSF Tau, these patterns were highly variable and only present in a small subset. Although DM1 may indeed represent a non-AD Tauopathy, the Tau-PET tracer used in this study was unable to detect an in vivo Tau DM1 signature in this small cohort. Interestingly, most DM1 participants presented with elevated plasma Nf-L and GFAP levels, suggestive of other, possibly related, central brain alterations which motivate further research. This pioneering study provides novel insights towards the potential relationship between biomarkers and neurocognitive deficits commonly seen in DM1.

## Introduction

Myotonic dystrophy type 1 (DM1) is a chronic, multisystemic, autosomal dominant neuromuscular disorder caused by an expanded CTG repeat in the *DMPK* gene. It is the most common form of adult muscular dystrophy worldwide. Neurologically, DM1 can manifest as muscle weakness, mild intellectual disability, and neurobehavioral changes with impairments in social cognition [19, 25, 32]. Underlying pathophysiological mechanisms are unknown but several neuropathological reports suggest that DM1 may be a Tauopathy where hyper and abnormally phosphorylated microtubule-associated Tau proteins accumulate in a topographic distribution similar to early stages of Alzheimer’s disease (AD) [2, 15]. Others have reported that brain Tau is alternatively spliced in DM1 [7, 11, 24] and CSF Tau levels may be modestly elevated [21, 31]. As reported, if Tau is mis-spliced and elevated in the CSF, it could be a pertinent biomarker for brain dysfunction and disease progression, with potential to demonstrate therapeutic benefit in clinical trials.

Structural neuroimaging abnormalities are well documented in prior studies of DM1 participants, including widespread gray matter loss in cortical and subcortical structures, ventriculomegaly, and white matter lesions [8]. White matter abnormalities [27], focal hypoperfusion [22] and reduced glucose metabolism in frontal, parietal and temporal lobes [20] have also been detected using diffusion tensor imaging, SPECT and FDG-PET. By contrast, there are no prior reports investigating Tau pathology in vivo in the brains of DM1 participants despite the fact that various Tau PET ligands are now readily available. One of the most studied Tau PET tracers, [18F]-AV-1451, was employed for this study. While many studies support the use of [18F]-AV-1451 to detect Tau pathology in participants with AD, its value for detecting non-AD Tauopathies is much less understood [12, 26]. DM1 is the only known disease characterized by the preferential aggregation of 0N3R Tau isoforms [2], and whether [18F]-AV-1451 binds to the conformation of Tau observed in DM1 [7, 28] is not yet known. A few studies have examined CSF biomarkers of neurodegeneration in DM1 and have reported fairly similar results. For instance, one study reported a statistically significant decrease in CSF Aβ 1-42 concentrations and increased levels of t-Tau in DM1 participants compared to healthy controls [31]. Similarly, while no statistically significant differences were reported, Peric et al. [21] showed that t-Tau and p-Tau levels were numerically higher and Aβ 1-42 was numerically lower in DM1 participants compared to healthy controls.

The goal of this exploratory study was thus to investigate Tau pathology in vivo in a small but well-characterized sample of DM1 participants using several biomarkers of neurodegeneration, including [18F]-AV-1451 Tau PET imaging, CSF and plasma biomarkers. We further aimed to study the relationship between pathology-associated biomarkers and severity of neurocognitive impairments in DM1, including social cognition deficits.

## Methods

### Participants

We initially recruited 19 participants with a genetic diagnosis of DM1 and two with Alzheimer's disease (AD) from neurology clinics at the largest academic tertiary care center in Eastern Quebec City, QC, Canada. The two participants with AD served as quality controls for Tau PET. Seven of the 19 DM1 participants withdrew from the study (3/7 changed their mind upon signing the informed consent, 2/7 were scheduled for surgery, and 2/7 were lost to follow-up); therefore, the final analysis sample reported herein included 12 DM1 participants and 2 AD participants. All participants provided written informed consent. This study was approved by the local Research Ethics Board.

### Neurocognitive and social cognition testing

Participants underwent cognitive screening using the Dépistage Cognitif de Québec^20^ (DCQ; http://dcqtest.org/), the Montreal Cognitive Assessment [18] (MoCA) and the Mini-Mental State Examination [6] (MMSE world and 100–7 versions). The DCQ is a 25-min cognitive screening test composed of five indexes (Memory, Visuospatial, Executive, Language, and Behavioral). It has been validated in both healthy participants and patients with atypical dementias. Assessment of social cognition was performed using the Behavioral Index of the DCQ. The Clinical Dementia Rating [17] (CDR) was used to grade the relative severity of dementia with scores from 0 (no impairment) to 3 (severe impairment). Participants were designated as cognitively impaired (CI) if their performance fell 2 standard deviations below the mean of the normative sample either on the MoCA or DCQ based on a separate, previously analyzed, normative sample [9]; those not meeting that criterion were considered to be cognitively unimpaired (CU).

### Tau PET imaging

During the PET scanning visit, each participant received an intravenous bolus infusion of ~ 10 mCi of [^18^F]-AV-1451, a radioligand which preferentially binds to neurofibrillary tangles. Sixty minutes post administration of the radioligand, participants were scanned for 30 min. Images were reconstructed applying scatter and attenuation correction and decay corrected to time of radioligand administration. Reconstructed images were also corrected for motion that might have occurred during scanning. Concomitant to the PET scan, participants also underwent a structural T1 MRI scan for the purpose of PET registration and region of interest (ROI) analysis. Standardized uptake value ratios (SUVR) images were generated using superior-cropped cerebellar gray matter as the reference region and average SUVR values were calculated in key ROIs, including composite ROIs corresponding to Braak I-II, Braak III-IV, and Braak V-VI regions [1, 13, 23].

### CSF biomarkers

CSF collection was performed through a lumbar puncture by an experienced neurologist. CSF concentrations of Aβ 1-42, Aβ 1-40, total-Tau, and phospho-Tau at threonine 181 were measured with the automated chemiluminescent enzyme-immunoassay (Lumipulse G 1200, Fujirebio Europe, Gent, Belgium). Analysis of the quality controls provided in the kits including three different levels of concentrations for each biomarker showed CV < 5% (2.3%, 4.8%, 5.0% and 4.0%, respectively). Standard CSF cut-offs provided by the manufacturer for distinguishing AD from other dementia and controls were 56.6 pg/mL for phospho-Tau, 400 pg/mL for total-Tau, 600 pg/mL for Aβ 1-42 and 6.9% for Aβ 1-42/Aβ 1-40 ratio [4].

### Plasma biomarkers

Blood samples were drawn before the lumbar puncture for quantification of plasma levels of amyloid Aβ 1-42 and Aβ 1-40, total Tau, Glial Fibrillary Acidic Protein (GFAP) and Neurofilament Light Chain (Nf-L). Plasma Aβ peptide assay was performed using the INNO-BIA kit (Fujirebio Europe NV, formely Innogenetics NV, Belgium), based on a multiplex xMAP technique with a LABScan-200 system (Luminex BV, The Netherlands). The interserial CV of Aβ 1-40 was between 7.1 and 7.6% (for levels of 216 and 106 pg/mL, respectively) and the interserial CV of Aβ 1-42 was between 3.0 and 9.4% (for levels of 93 and 198 pg/mL, respectively). The Neurology-4-plex assay was used (Lot 501858, Simoa technology, Quanterix Corporation, Lexington, MA, USA) on the SIMOA platform (Simoa technology, Quanterix Corporation, Lexington, MA, USA) to measure Nf-L, total-Tau and GFAP in plasma samples. This plasma biomarker analysis was performed by SpotToLab thanks to the clinical proteomic platform of Montpelier CHU headed by Professor Christophe HIRTZ (c-hirtz@chu-montpellier.fr). The limit of quantification for Nf-L was 0.38 pg/mL, total-Tau was 0.10 pg/mL and GFAP was 0.923 pg/mL. Analysis of the quality controls provided with the kit included low and high concentrations of Nf-L, total-Tau and GFAP with a CV lower than 10% (4%, 10% and 6%, respectively). To minimize the matrix effects, all samples were diluted fourfold with the diluent provided in the kit (phosphate buffer with bovine serum and heterophilic blocker solution) before analysis. Median plasma values of ten healthy donors indicated in the data sheet of the kit were: 8.58 pg/mL for Nf-L, 64.2 pg/mL for GFAP and 2.31 pg/mL for total-Tau, respectively; these were used as reference levels for our analysis to indicate abnormality.

### Statistical analyses

Descriptive results are expressed as means ± SD as well as the median and interval between the 1st and 4th quartile. Although this study is exploratory and primarily hypothesis generating, we performed preliminary analyses to compare biomarker profiles between CI vs. CU DM1 participants and to investigate associations between biomarker modalities. First, we performed uncorrected *t* tests on CSF and plasma biomarker levels between CI and CU participants. Second, we examined correlations between CSF and plasma biomarkers through Spearman rank correlation matrix using Graphpad Prism Software 8.4.2; correlations are represented on a heatmap. Spearman correlations between CSF and plasma biomarker levels were also explored (Stata SE 15.1). *P* values < 0.05 were considered significant.

### Data availability

Anonymized data will be made available upon any reasonable request from qualified investigators.

## Results

### Neurocognition and social cognition

Cognitive and social cognition testing scores are provided in Table 1, along with demographic information of the 12 DM1 participants and 2 AD participants. The two AD participants (#1 and #2) displayed a classic amnestic AD presentation on cognitive testing (MMSE and MoCA) with impaired episodic memory, visuospatial and executive skills. As expected, the CDR was higher for the two AD participants compared to the DM1 participants, suggesting greater functional impairment. Three (3/12) DM1 participants (#3, #8 and #19) were CI, as evidenced by lower mean scores on the MMSE (28/30 vs. 29/30), DCQ (79/100 vs. 90/100) and/or MoCA (23.3/30 vs. 29/30) relative to normative data previously reported [9]. Their performance on both the MMSE and MoCA indicated executive dysfunctions and the Social Cognition Index of the DCQ indicated significant social deficits, both defined by scores which fell 2 standard deviations below the mean of the normative sample. More specifically, three of the five indexes of the DCQ were impaired (Executive Index, Visuospatial Index, Social Cognition Index). CI participants were older than CU participants (53 vs. 46 years on average) but did not differ in number of years of education. Finally, there were no correlations between cognitive performance and blood repeat length.

**Table 1:** Demographics of the study participants

Variables	DM1 (*n* = 12)	Alzheimer’s disease (*n* = 2)*
Age (years; mean, sd)	47 (11.2)	67
Gender (F/M)	3/8	1/1
Education (years; mean, sd)	11.2 (1.2)	12
MMSE world (/30; mean, sd)	28.8 (1.9)	21
MMSE 100-7 (/30; mean, sd)	28.4 (2.1)	17
MoCA (/30; mean, sd)	26.6 (3.6)	13.5
DCQ Total (/100; mean, sd)	87.0 (9.9)	N/A
DCQ Behavioral Index (/20; mean, sd)	17.8 (3.0)	N/A
CDR (mean, sd)	0.3 (0.6)	2

*MMSE* Mini-Mental State Examination, *MoCA* Montreal Cognitive Assessment, *DCQ* Dépistage Cognitif de Québec (http://dcqtest.org/), *CSF* cerebrospinal fluid, *CDR* Clinical Dementia Rating

*Standard deviations are not reported for AD participants given sample size of two

### Tau PET imaging biomarkers

**Fig. 1:**
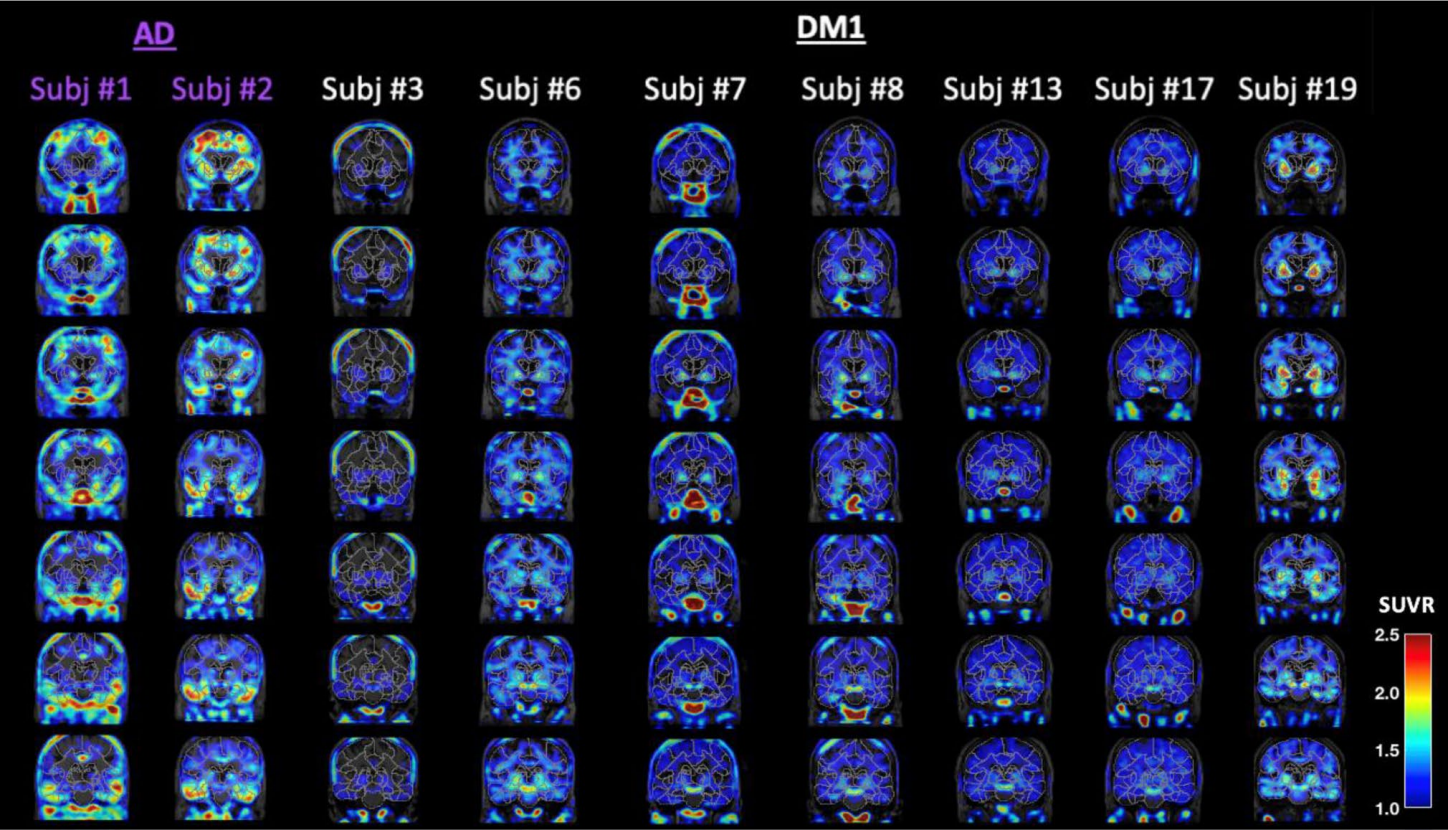
MK-6240 Tau PET images of AD (purple) and DM1 (white) participants. Coronal slices are displayed progressing anterior to posterior. SUVR images are overlaid on subject-specific T1 MRI images, as well as the outline of the subject-specific ROI atlas parcellation. SUVR scale is from 1 to 2.5

The two AD participants (#1 and #2) showed a typical AD-like pattern on Tau PET, including high retention across frontal, parietal, and temporal cortices and the medial temporal lobe; e.g. SUVR ≥ 1.5 (average of left and right) across composite ROIs corresponding to Braak stages I-V (see Fig. 1 and [Supplementary] Table S1). Of the three CI participants, one (#19) presented with increased Tau PET signal bilaterally in the medial temporal lobes (e.g. amygdala and hippocampus; Fig. 1). There was also prominent signal in striatum, but this is most likely attributable to off-target binding due to AV-1451 non-specificity [10]. SUVR values for this participant in the Braak I-II composite were 1.36 (right) and 1.50 (left), nearing that seen in the two AD cases ([Supplementary] Table S1). Patient #19 (aged 69 yo) was also the most severely cognitively impaired of the DM1 participants examined (DCQ = 61.5/100; MoCA = 17/30). Patient #8 (aged 45 yo), who was also mildly cognitively impaired (DCQ = 82.5/100; MoCA = 30/30), showed slight focal increase in Tau PET signal (e.g. right Braak I-II SUVR = 1.30). Tau PET in the last CI participant (#3) was unremarkable (see Fig. 1) despite mild cognitive deficits (DCQ = 82.5/100; MoCA = 27/30). The CU participants showed no uptake beyond that seen in the cerebellar reference region. Due to the limited signal present, statistical analysis comparing Tau PET SUVR between CI and CU participants was not performed.

### CSF biomarkers

Nine (9/12) DM1 participants completed the CSF portion of the study (see Table 2). CSF Total-Tau and phospho-Tau average concentrations were 256.44 ± 244 ng/mL and 38.88 ± 24.2 ng/mL, respectively. These concentrations were below the cut-off thresholds for an AD-like profile (i.e. > 400 pg/mL for total-Tau and > 56.6 pg/mL for phospho-Tau). Interestingly, CSF total-Tau and phospho-Tau levels were on average higher in CI participants (#3, #8 and #19) compared to CU participants (423 vs. 151.6 pg/mL and 64.3 vs. 26.42 pg/mL on average, respectively). Notably, patient #19 had the highest CSF total-Tau and phospho-Tau concentrations.

**Table 2:** CSF biomarkers in DM1 participants

Samples	Aβ 1-42 (pg/mL)	Aβ 1-40 (pg/mL)	Aβ 1-42/Aβ 1-40 Ratio (%)	Total-Tau (pg/mL)	Phospho-Tau (pg/mL)
#3	675	9897	6.8%	444	61.3
#4	1158	11,874	9.8%	283	53.4
#6	N/A	N/A	N/A	N/A	N/A
#7	510	5742	8.9%	135	21.7
#8	762	9136	8.3%	244	38.8
#10	635	7298	8.7%	111	20.1
#12	496	5744	8.6%	281	24.2
#13	N/A	N/A	N/A	N/A	N/A
#14	N/A	N/A	N/A	N/A	N/A
#15	335	4463	7.5%	115	17.4
#17	694	7823	8.9%	114	19.5
#19	223	8148	2.7%	581	92.8
Mean	609.78	7791.67	8%	256.44	38.80
Median	635.00	7823.00	9%	244.00	24.20
SD	270.12	2311.68	2%	165.14	25.79
1st quartile	496.00	5744.00	8%	115.00	20.10
Last quartile	694.00	8148.00	9%	281.00	38.80

Standard CSF cut-offs provided by the manufacturer for distinguishing Alzheimer’s disease from other dementia and controls are 56.6 pg/mL for phospho-Tau, 400 pg/mL for total-Tau, 600 pg/mL for Aβ 1-42 and 6.9% for Aβ 1-42/Aβ 1-40 ratio

Average CSF concentration of Aβ 1=42, Aβ 1-40 and Aβ 1-42/Aβ1-40 ratio in this DM1 series were 609.78 ± 270 ng/mL, 7791.67 ± 2311 ng/mL and 8% ± 2%, respectively. Therefore, on average, Aβ 1-42 did not reach the threshold for what is considered AD pathological levels (< 600 pg/mL) while Aβ 1-42/Aβ 1-40 ratio was slightly above an AD threshold (> 6.9%). The latter appears to be driven by relatively low levels of Aβ 1-40, which reflects overall production of amyloid peptides. Together, these data do not support presence of AD-type central amyloidopathy in DM1. When comparing DM1 CI participants to the CU DM1 participants, CSF amyloid markers Aβ 1-42 and Aβ 1-42/Aβ 1-40 ratio were lower in CI (553 vs. 638 pg/mL and 6.0 vs. 8.7% respectively), although this appears to be driven by DM1 patient #19. The mean level of Aβ 1-40 was higher in CI participants compared to CU participants (9060 vs. 7129 pg/mL).

### Plasma Biomarkers

All twelve DM1 participants completed the plasma portion of the study (see Table 3). Plasma biomarkers of neurodegeneration (Nf-L and Tau) and of glial cell lesions (GFAP) were higher in CI participants than CU participants (Nf-L: 50.91 vs. 13.04 pg/mL; Tau: 2.87 vs. 1.59 pg/mL; GFAP: 94.78 vs. 81.95 pg/mL). Of note, plasma Nf-L was markedly higher in patient #19 compared to all other participants (Table 3). Plasma Aβ 1-42/Aβ 1-40 ratio was also higher in CI participants than in CU participants (0.224 vs. 0.193 pg/mL). The Aβ 1-40 plasma concentrations were similar between CI and CU while Aβ 1-42 plasma concentrations were higher in CI (46.9 pg/mL) versus CU (38.48 pg/mL).

**Table 3:** Plasma biomarkers in DM1 participants

Participant samples	GFAP (pg/mL)	NF-L (pg/mL)	TAU (pg/mL)	Aβ 1-42 (pg/mL)	Aβ 1-40 (pg/mL)	Aβ 1-42/Aβ 1-40 Ratio
#3	99.75	39.08	3.78	51.6	242.7	0.213
#4	70.43	15.54	0.47	48.3	198.0	0.244
#6	61.42	20.84	1.40	58.5	267.3	0.219
#7	52.15	13.69	2.63	16.2	260.7	0.062
#8	92.61	12.80	2.89	42.6	173.1	0.246
#10	113.05	14.77	0.84	54.0	212.1	0.255
#12	N/A	N/A	N/A	49.5	193.5	0.256
#13	48.50	4.86	1.08	31.2	160.8	0.194
#14	122.42	20.18	3.12	42.0	188.1	0.223
#15	81.50	5.70	1.56	23.1	204.0	0.113
#17	106.17	8.72	1.60	34.5	192.9	0.179
#19	91.99	100.86	1.95	46.5	216.6	0.215
Mean	85.45	23.37	1.94	41.50	209.15	0.20
Median	91.99	14.77	1.60	44.55	201.00	0.22
SD	24.80	27.35	1.04	12.85	32.99	0.06
1st quartile	65.92	10.76	1.24	33.68	191.70	0.19
Last quartile	99.39	20.51	2.29	48.60	213.23	0.24

For reference, median plasma values of ten healthy donors indicated in the data sheet of the kit were: 8.58 pg/mL for Nf-L, 64.2 pg/mL for GFAP and 2.31 pg/mL for total-Tau, respectively

### Correlations between CSF and plasma biomarkers

**Fig. 2:**
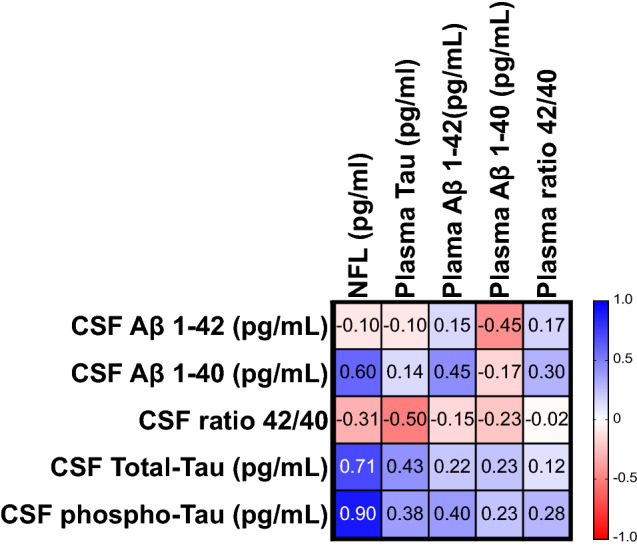
Correlations between CSF and plasma quantifications

Correlations were performed using the non-parametric Spearman rank correlation matrix as the cohort is small and the data do not follow a Gaussian distribution (see Fig. 2). Plasma Nf-L concentrations were shown to significantly correlate with CSF phospho-Tau concentration (*r* = 0.90; *p* = 0.005). Of note, this association remained significant even after removing participant #19, who had the highest levels of both biomarkers (*r* = 0.86, *p* = 0.024). Although the associations did not reach statistical significance, plasma Nf-L was also highly correlated with CSF total-Tau (*r* = 0.71) and with CSF Aβ 1-40 (*r* = 0.60). Correlations between Tau PET and fluid biomarkers were not performed due to low SUVR signal in all but one patient.

## Discussion

To our knowledge, this is the first study to explore Tau pathology in vivo in DM1 using Tau PET, CSF, plasma and neurocognitive biomarkers. Overall, the patterns and relationships between cognitive and biomarker profiles were heterogenous. Nevertheless, this study adds to a growing body of literature investigating the possible presence of Tau pathology in DM1. It further provides critical insight on sensitivity of tools to detect Tau pathology in vivo in these participants.

Three (3/12) DM1 participants were cognitively impaired, as evidenced by lower average scores on the DCQ and/or MoCA. Their neuropsychological profile indicated primary deficits in executive function and social cognition. Out of the three CI participants, two presented with some level of elevated Tau PET signal in the temporal lobes. The magnitude and extent of Tau PET signal was lower than that typically seen in AD participants, and only strongly present in one of the DM1 participants (#19). While this is consistent with the topological distribution of Tau pathology in neuropathological examination of post-mortem brain of DM1 participants [5, 7, 15], it also aligns with a typical early AD-like pattern with initial Tau deposits in the medial temporal lobes. The other DM1 participants did not show notably increased Tau PET signal relative to the reference region. Prior studies have suggested that [18F]-AV-1451 specifically binds to 3R/4R paired helical Tauopathy characteristic of AD, with relatively low affinity for other Tau filaments [12, 26]; to our knowledge, no studies have specifically tested whether 18F-AV-1451 or other available Tau PET tracers bind to the specific Tau conformation (principally made of 0N3R Tau isoforms) found in DM1 [2, 28]. Based on the current data showing little to no binding of [18F]-AV-1451 in the majority of DM1 participants in this sample, and only strong binding in the patient with possible concomitant AD, we conjecture that [18F]-AV-1451 either does not bind to DM1-specific Tau conformations, or that it is not sufficiently sensitive to detect the low levels of Tau pathology that are present in many or most DM1 participants. It is also possible that sub-populations of DM1 participants with higher levels of Tau pathology with inclusions made of both 3R and 4R Tau isoforms [7] that can be detected by [18-F]-AV-1451 exist, but additional studies with larger populations are needed to test that empirically. The potential value of other Tau PET tracers with different selectivity or specificity for DM1-specific Tau remains to be determined.

Relationships between CSF Tau and phospho-Tau with cognitive impairment were similarly heterogeneous. Two of the three CI DM1 participants (participants #3 and # 19) had elevated CSF total Tau and phospho-Tau above the AD cut-off levels. Levels of CSF Tau and phospho-Tau biomarkers were higher in participants that were CI than CU, but with a mean value lower than that typically observed in AD and more similar to other Tauopathies such as frontotemporal lobar degeneration [16]. In contrast to the CSF Tau findings, CSF amyloid levels were more homogenous. Apart from one patient (#19), all DM1 participants had normal CSF Aβ 1-42/Aβ 1-40 ratio levels suggesting the absence of AD-type central amyloidopathy as reported previously [15]. If post-mortem analysis of patient #19 were to confirm a diagnosis of concomitant AD, it is mostly likely that their abnormal CSF amyloid pattern is attributable to AD pathophysiology rather than DM1. At least two other studies have previously analyzed CSF amyloid and Tau levels in DM1 participants [21, 31]. In both studies Tau CSF levels in DM1 were greater than in healthy controls. Curiously, both studies also observed lower levels of CSF Aβ 1-42 in DM1 compared to controls; however, they did not analyze Aβ 1-42/A 1-β40 ratio, which is considered a more reliable measure of cerebral amyloidopathy [30]. This requires further replication given that amyloid deposits are seldom associated to neurofibrillary degeneration in DM1 brains [7, 15].

Plasma biomarkers of neurodegeneration (Nf-L and Tau), glial cell lesions (GFAP), and amyloid (Aβ 1-42, Aβ 1-40, and Aβ 1-42/Aβ 1-40 ratio) were all higher in CI participants than CU participants in this sample. Plasma Nf-L levels and GFAP levels were increased in most participants (8/10 and 7/10, respectively) relative to median levels in a healthy control sample, indicating that neuronal and glial alterations are possibly common to most DM1 participants; however, lack of a control sample in this study limits our interpretation. The gliosis in DM1 brain remains ill-defined [29]; increased levels of plasma GFAP could reflect an elevated gliosis process in DM1, but this requires further confirmation by post-mortem correlative analyses. Plasma Nf-L was strongly correlated with CSF phospho-Tau. A recent study [3] observed a similar correlation between plasma Nf-L and CSF Total-tau and phospho-Tau in cognitively normal participants with AD pathology defined by an AD CSF profile, while another study [14] did not. To our knowledge, this is the first study to investigate these fluid biomarker associations in DM1 participants. Correlation between CSF pTau and plasma Nf-L may reflect increased neuronal injury in both AD and DM1 but post-mortem analyses and replication in other DM1 cohorts are necessary to further corroborate this hypothesis.

This novel study adds to a growing body of research investigating central nervous system abnormalities in DM1, including Tau pathology. While some participants presented with abnormal Tau measured by Tau PET and/or CSF, these patterns were highly variable and only present in a small subset of our sample. Interestingly, the patient with the most severe cognitive impairment in our sample also demonstrated the most elevated Tau PET signal, the highest CSF phospho-Tau and Tau levels, and lowest CSF Aβ 1-42 and Aβ 1-42/ Aβ 1-40 levels, consistent with a typical AD biochemical profile. Whether this biomarker signal is related to more severe DM1 pathology or concomitant AD remains unknown and will necessitate further investigation. Indeed, one major limitation of this study is lack of post-mortem data to confirm biomarker sensitivity to DM1 Tau pathology, and to rule out other comorbidities, like AD, which may be contributing to both the observed cognitive impairment and abnormal Tau and/or amyloid biomarkers. A second major limitation is the small sample size; however, this study was designed to be exploratory and hypothesis generating. Post-mortem neuropathological examination (e.g., with autoradiographical confirmation of Tau-PET binding in DM1 brain tissue) and studies with larger samples of DM1 participants are necessary to further evaluate whether other Tau PET tracers and/or fluid biomarkers could represent pathological biomarkers in DM1. Studying various Tauopathies using multimodal biomarkers will help elucidate the pathological mechanisms of Tau and may help identify valuable biomarkers to support future therapies and clinical trials.
